# Successful Intubation Using a Cap-Assisted Colonoscope for Endoscopic Retrograde Cholangiopancreatography in Patients Undergoing Roux-en-Y Reconstruction

**DOI:** 10.3390/jcm12041353

**Published:** 2023-02-08

**Authors:** Kyong Joo Lee, Se Woo Park, Hyun Joo Jang, Da Hae Park, Jung Hee Kim, Jang Han Jung, Dong Hee Koh, Jin Lee

**Affiliations:** Division of Gastroenterology, Department of Internal Medicine, Hallym University Dongtan Sacred Heart Hospital, Hallym University College of Medicine, 7, Keunjaebong-gil, Hwaseong-si 18450, Republic of Korea

**Keywords:** endoscopic retrograde cholangiopancreatography, surgically altered anatomy, Roux-en-Y, jejuno-jejunostomy, colonoscope

## Abstract

Endoscopic retrograde cholangiopancreatography (ERCP) is challenging in patients undergoing Roux-en-Y (REY) reconstruction; although balloon-assisted enteroscopy is the first-line treatment, it is not always available considering equipment and expertise. We aimed to evaluate the feasibility of using a cap-assisted colonoscope as the primary approach for ERCP in REY reconstruction. We included 47 patients with REY who underwent ERCP using a cap-assisted colonoscope between January 2017 and February 2022. The primary outcome was intubation success for ERCP using a cap-assisted colonoscope during REY reconstruction. The secondary outcomes were cannulation success, procedure-related adverse events, and variables affecting successful intubation. Comparing side-to-side jejunojejunostomy (SS-JJ) and side-to-end jejunojejunostomy (SE-JJ) groups, the intubation success rate using a cap-assisted colonoscope in the SS-JJ group was higher than that in the SE-JJ group (34 of 38 (89.5%) vs. 1 of 9 (11.1%), *p* < 0.001). Successful intubation was achieved in 37 (97.4%) and 8 (88.9%) patients in the SS-JJ and SE-JJ groups, respectively, after applying the rescue technique using a balloon-assisted enteroscope for failed ERCP using only a colonoscope. No perforation occurred. Multivariable analysis showed that SS-JJ was a predictive factor for successful intubation (odds ratio [95% confidence interval] = 37.06 [3.91–925.56], *p* = 0.005). Usage of a cap-assisted colonoscope can be crucial for ERCP in patients undergoing REY reconstruction. Anatomically, SS-JJ can facilitate easy and accurate identification of the afferent limb and a highly successful ERCP using a cap-assisted colonoscope.

## 1. Introduction

**Figure 1:**
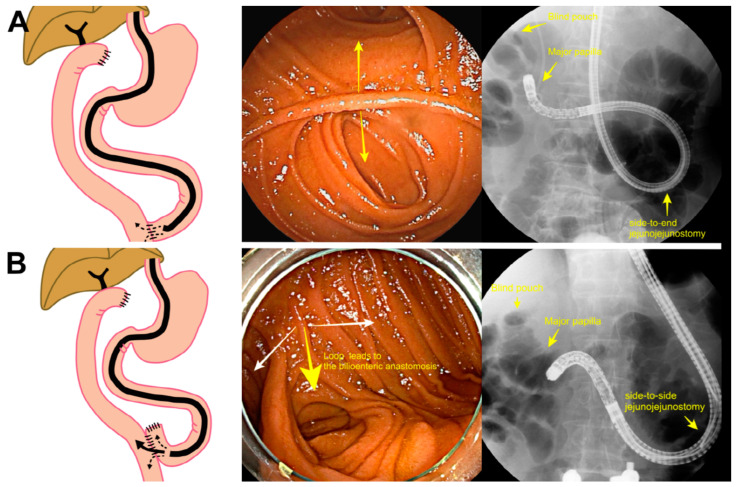
Type of jejunojejunostomy in Roux-en-Y reconstruction. (**A**) Schema of the operation procedure of side-to-end jejunojejunostomy; endoscopic view encountering two-pronged lumens at the jejunojejunostomy; fluoroscopic image showing the short-type double-balloon enteroscope in the afferent loop. (**B**) Schema of the operation procedure of side-to-side jejunojejunostomy; endoscopic view encountering three-pronged lumens at the jejunojejunostomy; fluoroscopic image showing the cap-assisted colonoscope in the afferent loop.

Endoscopic retrograde cholangiopancreatography (ERCP) in patients with surgically altered anatomy (SAA) has been technically challenging in terms of successful intubation and sequential ERCP-related interventions, including selective cannulation [1,2]. Successful intubation is defined as the endoscope reaching the end of the afferent limb; it is essential but challenging, especially in patients who undergo Roux-en-Y (REY) reconstruction [3]. First, identifying the afferent limb is critical for successful intubation; however, there is little conclusive information regarding the loop that is advanced to the bile duct (papilla or bilioenteric anastomosis). Recently, Tsutsumi et al. classified jejunojejunostomy (JJ) reconstruction in REY anastomosis as side-to-end jejunojejunostomy (SE-JJ) (Figure 1A) and side-to-side jejunojejunostomy (SS-JJ) (Figure 1B) considering the ERCP procedure [4]. They demonstrated that SE-JJ generally has a long bidirectional loop, but the loop leading to the papilla or bilioenteric anastomosis cannot be easily discriminated. Therefore, endoscopists first selected a random loop to reach the papilla or bilioenteric anastomosis in the JJ. If the scope was inserted into the wrong loop, they withdrew it until the JJ and advanced the other loop from the JJ. Endoscopists encounter three loops at the anastomosis site in patients undergoing SS-JJ. Among the three loops, Tsutsumi et al. recommended advancing the scope to the middle loop, which can help reach the papilla or bilioenteric anastomosis successfully. Based on the classifications, they successfully intubated the papilla or bilioenteric anastomosis in 84% of 59 patients undergoing SE-JJ and 100% of 31 patients undergoing SS-JJ using double-balloon enteroscope (DBE). Therefore, the authors concluded that, with SS-JJ, it is anatomically easy to accurately identify the afferent limb without hesitation owing to uncertainty regarding the lumen that leads to papilla or bilioenteric anastomosis, and it is highly successful in reaching the bile duct.

Most studies regarding ERCP in REY reconstruction provided excellent outcomes using a balloon-assisted enteroscope, but not using an easily accessible colonoscope that does not require additional instruments, such as an overtube-equipped balloon. Although cap-assisted colonoscopy has also been increasingly used for ERCP in Billroth II gastrectomy, it has limitations in patients undergoing REY reconstruction because of the absence of anchoring with an overtube or balloon and the relatively short scope length. Recent studies demonstrated that intubation of the papilla was successful only in 50–68.8% of cases when the colonoscope was used alone [3,5]. There is insufficient evidence focussing on the role of cap-assisted colonoscopy in ERCP in patients undergoing REY reconstruction. Therefore, to provide more evidence for this, we aimed to evaluate the feasibility and safety of cap-assisted colonoscopes and determine the variables that affect intubation success for ERCP in patients undergoing REY reconstruction.

## 2. Materials and Methods

### 2.1. Patients

This single-center retrospective study was conducted at Hallym University Dongtan Sacred Heart Hospital. This study included patients who underwent ERCP for REY reconstruction. Patients who underwent ERCP using other scopes instead of a cap-assisted colonoscope on the first attempt were excluded. Demographic, endoscopic, and clinical data were extracted from a computerized clinical information system from January 2017 to February 2022. The protocol adhered to the principles of the Declaration of Helsinki and the institutional review board of the Hallym Dongtan Sacred Hospital approved this study (IRB file no: 2022-04-008). The need for informed consent was waived because of the study’s retrospective nature.

### 2.2. Endoscopic Procedures for ERCP

All procedures were performed at the first attempt with a regular forward-viewing colonoscope (EC-760R-V/L, Fujifilm Medical Systems, Tokyo, Japan) by an experienced endoscopist (S.W.P.) who has worked on more than 700 patients per year, with ERCP conducted under a well-established standard protocol. The colonoscope consisted of a working length of 169 cm and a working channel diameter of 3.8 mm. If the initial attempt using a cap-assisted colonoscope failed, the rescue technique was attempted using long-type DBE (EN-580T, Fujifilm Medical Systems, Tokyo, Japan) with a working length of 200 cm and a working channel diameter of 3.2 mm or short-type DBE (EI-580BT, Fujifilm Medical Systems, Tokyo, Japan) with a working length of 155 cm and a working channel diameter of 3.2 mm. The distal end diameters of colonoscope and DBE are 12 mm and 9.4 mm, respectively. Furthermore, the ERCP devices used were not limited to any specific type. The contrast medium was only injected when the endoscopist confirmed if selective deep cannulation of the target duct was achieved using a guidewire. Additionally, endoscopic papillary balloon dilation was performed to reduce the risk of post-ERCP bleeding in patients with coagulopathy or a high bleeding tendency.

### 2.3. Definitions and Outcome Measurements

The primary outcome was the technical success of intubation for ERCP using a cap-assisted colonoscope in REY reconstruction. The secondary outcomes were the cannulation success rate, procedure-related adverse events, and identification of variables that affected successful intubation using a logistic regression model.

Successful intubation is defined as reaching the papilla or bilioenteric anastomosis, while successful cannulation is defined as the successful deep cannulation of the bile duct or pancreatic duct based on successful intubation [6]. The intubation time was defined as the time from the insertion of the colonoscope through the mouth to the time of reaching the papilla or bilioenteric anastomosis. Cannulation time was defined as the time interval from endoscopically appropriate effacement of the papilla or bilioenteric anastomosis to fluoroscopically deep cannulation of the guidewire. Furthermore, difficult cannulation was defined as cannulation that lasted more than 5 min, cannulation attempts of more than five times, or inadvertent pancreatic duct cannulation of more than three times [7].

Regarding adverse events associated with ERCP, post-ERCP pancreatitis (PEP) was defined as new-onset or deteriorated abdominal pain with an elevated serum amylase level to over threefold of the upper normal limit for 24 h after a procedure requiring hospitalization for at least 2 days. Post-ERCP bleeding was considered when the patient presented with melena or hematemesis, at least a 2 g/dL decrease in hemoglobin, or a need for blood transfusion [6].

### 2.4. Statistical Analysis

Categorical variables were presented as frequencies with proportions and compared using the χ^2^ test, while continuous variables were presented as standard deviation (SD) (or median with interquartile range (IQR)) and compared using Student’s *t*-test. Predictive factors for successful intubation were identified using univariate and multivariate logistic regression analyses. Odds ratios (ORs) and 95% confidence intervals (CIs) were estimated using a multivariate logistic regression model, adjusted for all variables. All reported *p*-values were two-sided and statistical significance was set at *p* < 0.05. All statistical analyses were conducted using R statistical software.

## 3. Results

### 3.1. Baseline Characteristics

**Figure 2:**
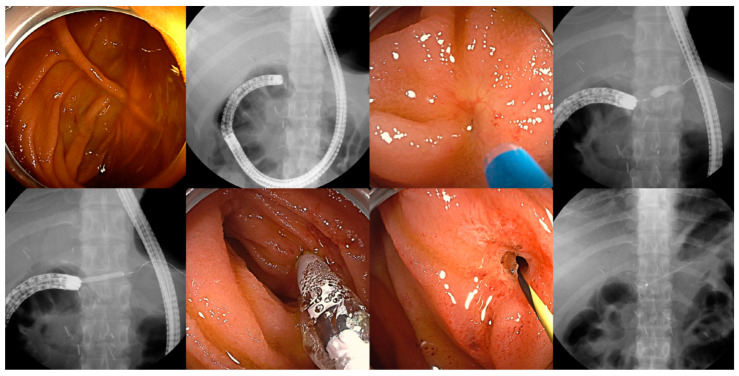
The procedure of endoscopic retrograde cholangiopancreatography using a cap-assisted colonoscope in a patient with pancreaticojejunostomy (PJ) stricture after pylorus-preserving pancreatoduodenectomy with Roux-en-Y reconstruction and side-to-side jejunojejunostomy. After reaching the end of the afferent loop, the small hole indicating the PJ site was found. Balloon dilatation of the PJ stricture was performed. Subsequently, a plastic stent was placed in the main pancreatic duct for the prevention of recurrence of stricture.

A total of 47 patients who underwent ERCP for REY reconstruction using a cap-assisted colonoscope were included (Table 1). The mean age of the patients was 66.6 years and 61.7% of the patients were male. The main surgeries included 21 cases of subtotal gastrectomy, 12 cases of pylorus-preserving pancreaticoduodenectomy, 6 cases of bile duct resection with cholecystectomy, 4 cases of total gastrectomy, 3 cases of hepatectomy with bile duct resection, and 1 case of Whipple operation. JJ reconstructions were classified as REY with SE-JJ (19.1%) and REY with SS-JJ (80.9%). The indications for ERCP were benign strictures (48.9%) (Figure 2), malignant strictures (31.9%), choledocholithiasis (14.9%) (Video S1 and Figure S1), and bile leakage after surgery (4.3%).

### 3.2. Technical and Clinical Outcomes According to the Anastomosis Type

The outcomes of ERCP procedures are summarized in Table 2. The total intubation success rate was 95.7%. Using a cap-assisted colonoscope, the total intubation success rate was 74.4%. The intubation success rate using the cap-assisted colonoscope was significantly higher in the SS-JJ group than in the SE-JJ group (89.5% vs. 11.1%, *p* < 0.001). DBE was applied as a rescue technique for cases of failed intubation using an initial cap-assisted colonoscope. The intubation success rates for DBE were 87.5% in the SE-JJ group and 75% in the SS-JJ group. The total cannulation success rate was not different between the groups. The intubation time was significantly shorter in the SS-JJ group than in the SE-JJ group (9.9 ± 8.7 vs. 19.8 ± 11.7 min, respectively, *p* = 0.018). However, the total procedure and cannulation times did not differ between the groups.

Total adverse events occurred in 6.4% of the patients. PEP was identified among three patients in the SS-JJ group, although no statistical differences were found (*p* = 0.859). All patients recovered with conservative care, such as fluid therapy. No major adverse events, such as significant post-ERCP bleeding or perforation, occurred in either group. Additionally, no significant difference in the length of hospital stay was found between the groups.

### 3.3. Predictive Factors Associated with Successful Intubation Using a Cap-Assisted Colonoscope

Predictive factors associated with successful intubation using a cap-assisted colonoscope were evaluated (Table 3). In the univariate analysis, an intact stomach (OR 0.17; 95% CI 0.03–0.70, *p* = 0.021) and SS-JJ (OR 68.00; 95% CI 9.34–1452.28, *p* < 0.001) were identified as predictive factors for successful intubation. Multivariate analysis also showed that SS-JJ was a predictive factor for successful intubation even after adjusting for potentially confounding variables, including age, sex, bilioenteric anastomosis, and presence of an intact stomach (OR 37.06; 95% CI 3.91–925.56, *p* = 0.005).

## 4. Discussion

This study evaluated the clinical outcomes and adverse events associated with ERCP using a cap-assisted colonoscope in patients with REY reconstruction. Furthermore, it demonstrated that using a cap-assisted colonoscope as the first attempt for ERCP in patients with REY is technically feasible and safe, with an overall intubation success rate of 74.4% and no major adverse events such as perforation or mortality. The successful intubation rate was increased to 89.5% in cases of REY with SS-JJ using only a colonoscope without an overtube or using an enteroscope that is difficult to handle. Furthermore, regression analysis confirmed that REY with SS-JJ was the most powerful predictor for successful intubation using a cap-assisted colonoscope for ERCP.

Endoscopists should understand the reconstruction anatomy based on preprocedural imaging modalities and make a blueprint regarding adequate scope selection and driving techniques before starting ERCP in patients with REY. Regarding appropriate scope selection, ERCP using a conventional side-view duodenoscope has been reported to be unsatisfactory in patients with REY reconstruction because of unpredictable altered anatomy, a longer afferent limb, sharp angulation of the jejunojejunal anastomosis, and an opposite direction of the papilla [8,9]. Therefore, more advanced endoscopes, including long- and short-type DBE and, most recently, a through-the-scope anchoring balloon catheter system, have been applied to these patients [10]. Among these, long-type DBE is safe and efficient for facilitating ERCP, with a 90% pooled intubation success rate in patients with SAA in the early stage of the DBE era [11]. However, conventional accessories dedicated for ERCP cannot be used through long-type DBE, with a long working length of 200 cm; therefore, conversion from long-type DBE to a short scope under remaining overtube should be considered for therapeutic ERCP (Figure S2) [5]. To overcome this limitation, the use of short-type DBE with a 155 cm working length and a 3.2 mm enlarged working channel has been announced [12,13,14]. In contrast, a colonoscope is easily accessible and has a larger working channel diameter (3.8 mm); therefore, most standard ERCP accessories can be easily used with this scope. Thus, efficiency can be maximized using only a standard colonoscope along with expensive medical equipment and including additional experts for DBE. We reached the papilla or bilioenteric anastomosis in 74.4% of the patients using a colonoscope alone. This is higher than the success rate reported in a previous trial that evaluated the use of a conventional colonoscope in patients with REY reconstruction [5,15]. Furthermore, successful cannulation was achieved in most patients, although it took longer in some. The higher success rate of cannulation might be attributed to the direction of the working channel, which was located at the scope’s five-to-six-o’clock position. Cannulation is easier if the scope rotates the papilla to the en face view with the endoscopic working channel [2].

Although the length of the afferent limb is different depending on the type of reconstruction, REY reconstruction usually involves a long afferent limb >100 cm as well as adhesion formation and sharp angulation of anastomosis [2,16]. In our study, intubation was significantly more successful in patients who underwent SS-JJ reconstruction than in those who underwent SE-JJ reconstruction. Tsutsumi et al. also reported that, while using DBE, the success intubation rates were higher in the SS-JJ group (100%) compared with the SE-JJ group (84%) (*p* = 0.039) [4]. This can be explained by understanding the surgical anatomy. Tokuhara et al. described that the Y-anastomosis fixed loosely in the abdominal cavity and Treitz ligament is long in patients with REY [1]. As shown in Figure 1A, two-pronged lumens face the anastomosis in patients with SE-JJ. The endoscopist approaches one lumen; if this fails, insertion into the other lumen is attempted. Owing to the afferent limb’s acute angulation, a long alpha loop is usually formed by the force applied at the Y-anastomosis. In contrast, three-pronged lumens are identified at the anastomosis in patients with SS-JJ; advancing across the middle lumen can approach bilioenteric anastomosis [4]. As the angulation at anastomosis is less sharp compared with SE-JJ, a loop is rarely formed (Figure 1B) [17].

Loop formation is problematic for ERCP in REY using only a colonoscope unsupported by anchoring devices such as an over-tube or balloon. The following five fundamental and mechanical solutions are used to solve this problem: (1) advance the scope with simultaneously minimal but continuous twisting of the scope body, (2) minimize air inflation, (3) prevent excessive pushing against the jejunal wall, (4) apply hand pressure on the patient’s belly, and (5) change the patient’s position. Furthermore, the stiffness of the scope itself resists bending in a spring-like manner in well-developed modern scopes optimized to be pushed through the jejunum [18].

Our study is the first to focus on the role of cap-assisted colonoscopy in ERCP in patients with REY reconstruction with a relatively large study population. Nevertheless, this study has several limitations. First, the study was retrospective and was conducted at a single institution. Second, a bias may have occurred considering the expertise and learning curve of the cap-assisted colonoscope in patients undergoing REY reconstruction, as only one endoscopist participated in the study. Third, the number of patients who underwent SE-JJ was smaller than those who underwent SS-JJ. Finally, cap-assisted colonoscopy was first attempted in REY patients for ERCP, so there was no control group.

## 5. Conclusions

A cap-assisted colonoscope for ERCP in patients with REY is effective and safe. Therefore, it is reasonable as the first attempt for ERCP in REY patients, especially with SS-JJ, although it is not always possible to reach the target site, and subsequent treatments are technically difficult. There is room for improvement; therefore, a large prospective randomized study is required to confirm our results.

## Figures and Tables

**Table 1:** Baseline characteristics of the patients.

Variable	Patients Underwent REY *n* = 47
Age, years, mean ± SD	66.6 ± 17.8
Sex, *n* (%)	
Male	29 (61.7%)
Female	18 (38.3%)
Surgery, *n* (%)	
BDR with cholecystectomy	6 (12.8%)
Hepatectomy with BDR	3 (6.4%)
PPPD	12 (25.5%)
Subtotal gastrectomy	21 (44.7%)
Total gastrectomy	4 (8.5%)
Whipple’s operation	1 (2.1%)
Intact stomach	21 (44.7%)
Type of anastomosis, *n* (%)	
REY SE-JJ	9 (19.1%)
REY SS-JJ	38 (80.9%)
Indication of ERCP, *n* (%)	
Malignant stricture	15 (31.9%)
Benign stricture	23 (48.9%)
Choledocholithiasis	7 (14.9%)
Bile leakage after surgery	2 (4.3%)

REY, Roux en Y reconstruction; SD, standard deviation; BDR, bile duct resection; PPPD, pylorus preserving pancreaticoduodenectomy; SE, side to end; JJ, jejuno-jenunostomy; SS, side to side; ERCP, endoscopic retrograde cholangiopancreatography.

**Table 2:** Technical and clinical outcomes according to the type of anastomosis.

Variable	REY with SE-JJ *n* = 9	REY with SS-JJ *n* = 38	*p*-Value
Total intubation success, *n* (%)	8 (88.9%)	37 (97.4%)	0.830
Cap-assisted colonoscope	1/9 (11.1%)	34/38 (89.5%)	<0.001
Rescue techniques	7/8 (87.5%)	3/4 (75%)	
Long type DBE	4/5	1/2	
Short type DBE	2/2	2/2	
Conversion from long to short DBE	1/1	0	
Total cannulation success, *n* (%)	7 (77.8%)	33 (86.8%)	0.868
Total procedure time, min, mean ± SD	30.4 ± 17.6	28.3 ± 17.3	0.745
Intubation time, min, mean ± SD	19.8 ± 11.7	9.9 ± 8.7	0.018
Cannulation time, min, mean ± SD	0.8 ± 0.3	3.8 ± 8.6	0.051
Difficult cannulation ^a^, *n* (%)	5 (55.6%)	14 (36.8%)	0.515
Rescue infundibulotomy, *n* (%)	0 (0.0%)	2 (5.3%)	>0.999
Endoscopic sphinctereotomy, *n* (%)	0 (0.0%)	2 (5.3%)	>0.999
EPBD, *n* (%)	0 (0.0%)	12 (31.6%)	0.126
Biliary stent placement, *n* (%)	0 (0.0%)	14 (36.8%)	0.077
Plastic stent	0 (0.0%)	10 (26.3%)	0.193
Metal stent	0 (0.0%)	4 (10.6%)	
Pancreatic stent placement, *n* (%)	0 (0.0%)	3 (7.9%)	0.910
Perforation	0 (0.0%)	0 (0.0%)	
Post-ERCP bleeding	0 (0.0%)	0 (0.0%)	
Post-ERCP pancreatitis, *n* (%)	0 (0.0%)	3 (7.9%)	0.910
Severity of Post-ERCP pancreatitis, *n* (%)			0.859
Mild	0 (0.0%)	1 (2.6%)	
Moderately severe	0 (0.0%)	1 (2.6%)	
Severe	0 (0.0%)	1 (2.6%)	
Mortality	0 (0.0%)	0 (0.0%)	
Length of hospital stay, day, mean ± SD	3.1 ± 2.2	4.1 ± 4.7	0.343

^a^ Difficult cannulation was defined as cannulation attempts more than five times, long cannulation time more than five minutes, or unintentional pancreatic duct cannulation more than three times. REY, Roux en Y reconstruction; DBE, Double balloon enteroscope; SD, standard deviation; EPBD, endoscopic papillary balloon dilation; ERCP, endoscopic retrograde cholangiopancreatography.

**Table 3:** Prediction of successful intubation using colonoscope.

Variable	*n*	Success, *n* (%)	Univariable Analysis		Multivariable Analysis	
			OR (95% CI)	*p*-Value	OR (95% CI)	*p*-Value
Age						
≤65 years	15	11 (73.3)	1		1	
>65 years	32	27 (84.4)	2.89 (0.74–11.67)	0.127	1.54 (0.09–54.30)	0.777
Sex						
Female	18	16 (88.9)	1		1	
Male	29	22 (75.9)	0.44 (0.09–1.79)	0.279	0.61 (0.04–6.97)	0.686
Bilo-enteric anastomosis						
No (Presence of papilla)	30	23 (76.7)	1		1	
Yes	17	15 (88.2)	0.85 (0.19–3.27)	0.813	0.22 (0.01–3.39)	0.324
Intact stomach						
No	26	24 (92.3)	1		1	
Yes	21	14 (66.7)	0.17 (0.03–0.70)	0.021	0.13 (0.01–2.04)	0.181
Type of JJ anastomosis						
SE-JJ	7	1 (14.3)	1		1	
SS-JJ	39	36 (92.3)	68.00 (9.34–1452.28)	<0.001	37.06 (3.91–925.56)	0.005

OR, odds ratio; CI, confidence interval; JJ, jejuno-jenunostomy; SE, side to end; SS, side to side.

## Data Availability

The datasets generated during and/or analyzed during the current study are available from the corresponding author upon reasonable request.

## Supplementary Materials

The following supporting information can be downloaded at https://www.mdpi.com/article/10.3390/jcm12041353/s1. Video S1: Foreign body removal using a cap-assisted colonoscope for endoscopic retrograde cholangiopancreatography (ERCP) in patients with Roux-en-Y (REY) and side-to-side jejunojejunostomy. ERCP was carried out using a cap-assisted colonoscope in a patient who underwent pylorus-preserving pancreaticoduodenectomy with REY reconstruction and side-to-side jejunojejunostomy (modified Child surgery). After the passage of the scope through gastro-jejunostomy, we encountered three-pronged lumens identified at the side-to-side jejunojejunostomy. Among three lumens, the scope is advanced across the middle lumen. Subsequently, the scope could successfully reach the bilioenteric anastomosis after several times of shortening the loop. After effacement of the bilioenteric anastomosis under a stable scope position, we observed large amounts of biliary cast encasing the surgical stent placed during operation as a cause of biliary obstruction in the bilioenteric anastomosis. Finally, we completely removed foreign bodies using forceps several times. Figure S1: The procedure of endoscopic retrograde cholangiopancreatography in a patient with common bile duct (CBD) stones after subtotal gastrectomy with Roux-en-Y reconstruction and side-to-side jejunojejunostomy. After reaching the end of the afferent loop using a cap-assisted colonoscope, balloon dilatation was performed. Subsequently, multiple CBD stones were completely removed using a balloon and basket catheter. Figure S2: The endoscope exchange technique procedure in a patient with bile duct resection with Roux-en-Y reconstruction and side-to-end jejunojejunostomy. First, a long-type double-balloon enteroscope (DBE) equipped with a short-type over-tube reached the end of the afferent loop. The long-type DBE was removed through the indwelling over-tube while its balloon was inflated. Then, the short-type DBE was inserted through the over-tube and balloon dilatation was performed for the hepaticojejunostomy stricture. Subsequently, multiple intrahepatic duct stones were completely removed with a balloon catheter.
