# Supplementary material for: Successful Intubation Using a Cap-Assisted Colonoscope for Endoscopic Retrograde Cholangiopancreatography in Patients Undergoing Roux-en-Y Reconstruction
Source: J Clin Med. 2023 Feb 8;12(4):1353. doi: 10.3390/jcm12041353 (PMC9966074; doi:10.3390/jcm12041353)
Supplement: Supplementary file 1 [file jcm-12-01353-s001.zip › Supplementary Figures.pdf]

Supplementary Figures

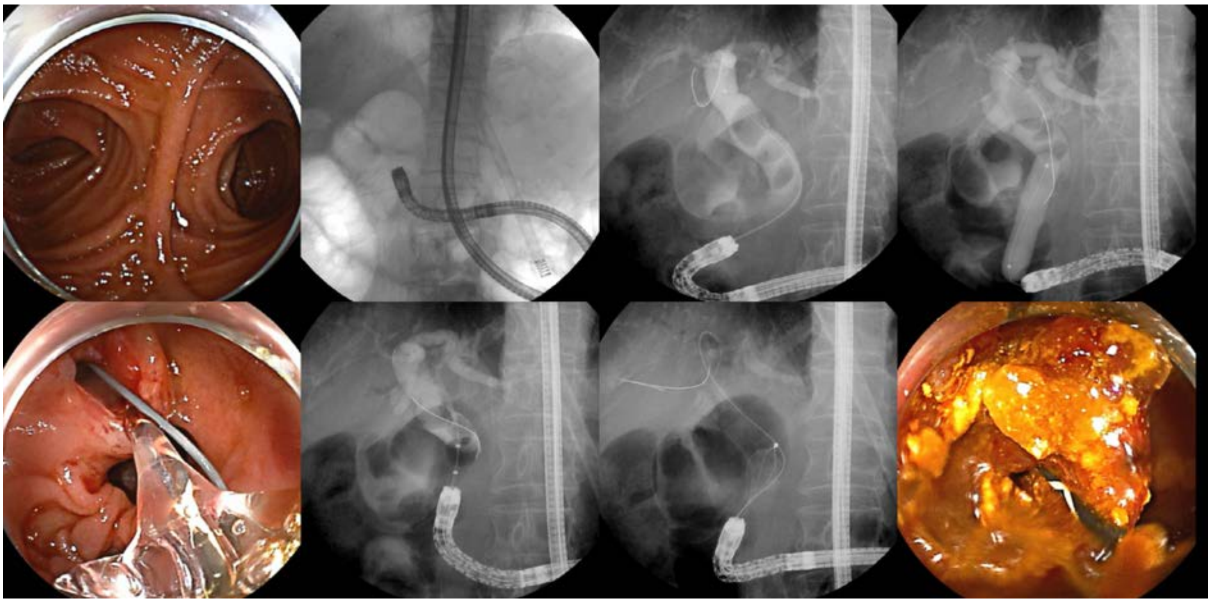

Figure S1. The procedure of endoscopic retrograde cholangiopancreatography in a patient with common bile duct (CBD) stones after subtotal gastrectomy with Roux-en-Y reconstruction and side-to-side jejunojejunostomy. After reaching the end of the afferent loop using a cap-assisted colonoscope, balloon dilatation was performed. Subsequently, multiple CBD stones were removed using a balloon and basket catheter completely.

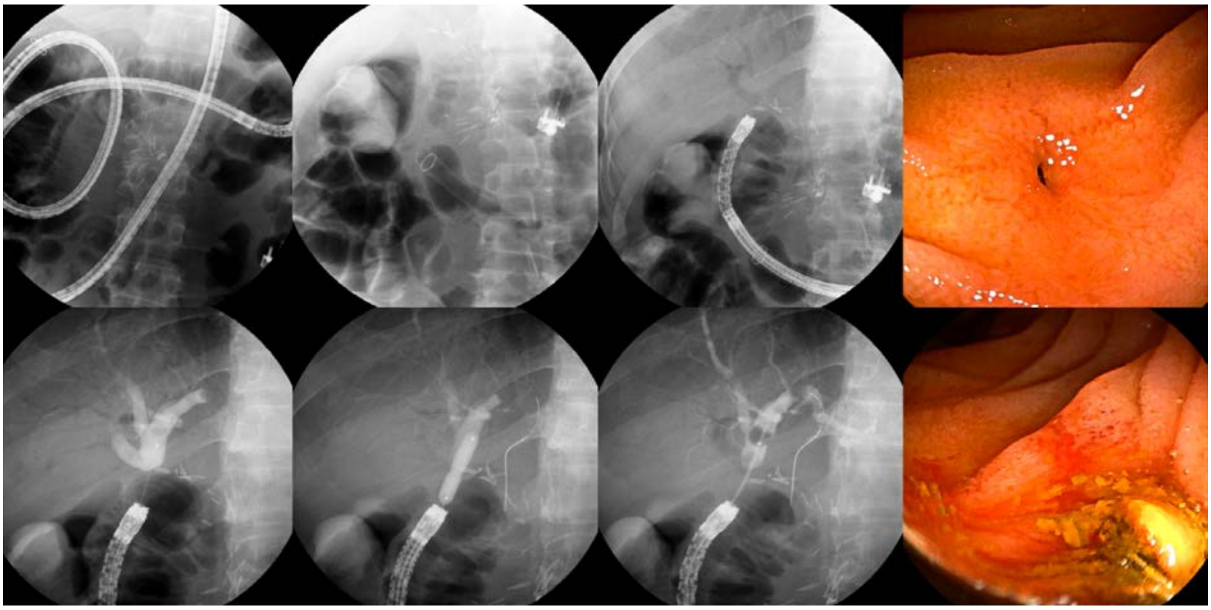

Figure S2. The endoscope exchange technique procedure in a patient with bile duct resection with Roux-en-Y reconstruction and side-to-end jejunojejunostomy. First, a long-type double-balloon enteroscope (DBE) equipped by a short-type overtube reached the end of the afferent loop. The long-type DBE was removed through the indwelling overtube while its balloon is inflated. Then, the short-type DBE was inserted through the overtube, and balloon dilatation was performed for hepaticojejunostomy stricture. Subsequently, multiple intrahepatic duct stones were removed with a balloon catheter completely.
